# The role of NHE3 (Slc9a3) in oxalate and sodium transport by mouse intestine and regulation by cAMP

**DOI:** 10.14814/phy2.14828

**Published:** 2021-04-27

**Authors:** Christine E. Stephens, Jonathan M. Whittamore, Marguerite Hatch

**Affiliations:** ^1^ Department of Pathology, Immunology, and Laboratory Medicine College of Medicine University of Florida Gainesville FL USA

**Keywords:** cAMP, intestine, ion transport, NHE3, oxalate, sodium

## Abstract

Intestinal oxalate transport involves Cl^−^/HCO_3_^−^ exchangers but how this transport is regulated is not currently known. NHE3 (Slc9a3), an apical Na^+^/H^+^ exchanger, is an established target for regulation of electroneutral NaCl absorption working in concert with Cl^−^/HCO_3_^−^ exchangers. To test whether NHE3 could be involved in regulation of intestinal oxalate transport and renal oxalate handling we compared urinary oxalate excretion rates and intestinal transepithelial fluxes of ^14^C‐oxalate and ^22^Na^+^ between NHE3 KO and wild‐type (WT) mice. NHE3 KO kidneys had lower creatinine clearance suggesting reduced GFR, but urinary oxalate excretion rates (µmol/24 h) were similar compared to the WT but doubled when expressed as a ratio of creatinine. Intestinal transepithelial fluxes of ^14^C‐oxalate and ^22^Na^+^ were measured in the distal ileum, cecum, and distal colon. The absence of NHE3 did not affect basal net transport rates of oxalate or sodium across any intestinal section examined. Stimulation of intracellular cAMP with forskolin (FSK) and 3‐isobutyl‐1‐methylxanthine (IBMX) led to an increase in net oxalate secretion in the WT distal ileum and cecum and inhibition of sodium absorption in the cecum and distal colon. In NHE3 KO cecum, cAMP stimulation of oxalate secretion was impaired suggesting the possibility of a role for NHE3 in this process. Although, there is little evidence for a role of NHE3 in basal intestinal oxalate fluxes, NHE3 may be important for cAMP stimulation of oxalate in the cecum and for renal handling of oxalate.

## Graphical abstract

A high urinary oxalate excretion rate is a risk factor for developing kidney stones, however, the intestine can serve as an extra‐renal pathway for excretion of oxalate from the body. Here we show that NHE3, a sodium‐hydrogen exchange protein, is required for cAMP stimulated oxalate secretion in the murine cecum. However, we found no evidence for the involvement of NHE3 in oxalate transport across either the murine ileum or distal colon.

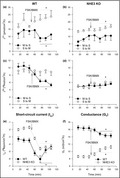

## 1 INTRODUCTION

High rates of oxalate excretion in the urine (hyperoxaluria) are a risk factor for kidney stones (Asplin, 2002; Coe et al., 2005; Sakhaee et al., 2012). The intestine makes a major contribution to oxalate homeostasis, not only from the absorption of dietary oxalate but also as a valuable extrarenal excretion pathway. Urinary oxalate excretion can be reduced in association with enhanced intestinal secretion in chronic renal failure, colonization with the gut bacteria *Oxalobacter formigenes*, and metabolic acidosis in rats (Hatch, 2014; Hatch et al., 2011; Kaufman et al., 2008; Robijn et al., 2011; Whittamore & Hatch, 2015, 2016). This has generated interest in understanding the mechanism(s) responsible for intestinal epithelial oxalate secretion, identifying the transporters responsible and how they are regulated. So far, two apical chloride/bicarbonate (Cl^−^/HCO_3_^−^) exchangers PAT1 (putative anion transporter 1, Slc26a6) and DRA (downregulated in adenoma, Slc26a3) have been shown to be involved in the secretion (Freel et al., 2006; Jiang et al., 2006) and absorption of oxalate, respectively (Freel et al., 2013). Despite this, we still do not understand how intestinal oxalate transport is regulated through these transporters. In the mammalian intestine, PAT1 and DRA play a role in electroneutral NaCl absorption, which involves coupled NHE3 mediated Na^+^/H^+^ exchange and Cl^−^/HCO_3_^−^ exchange supporting fluid absorption (Binder et al., 1987; Knickelbein et al., 1985; Musch et al., 2009; Seidler et al., 2008; Walker et al., 2008; Xu et al., 2018). It could be that the regulation of oxalate transport through PAT1 and DRA is related to their functional and physical relationship with the sodium‐hydrogen antiporter 3 (NHE3, SLC9A3).

A variety of neurohumoral inputs modify and coordinate Na^+^/H^+^ and Cl^−^/HCO_3_^−^ exchange in a segment‐specific manner mediated through the intracellular secondary messengers cAMP, cGMP and Ca^2+^ (Chen et al., 2010; Field, 2003; Kato & Romero, 2011). cAMP stimulates oxalate secretion in the rabbit's small and large intestine (Freel et al., 1998; Hatch, Freel, & Vaziri, 1993, 1994b), and recently, stimulation of cAMP production was shown to dramatically enhance apical PAT‐1‐mediated oxalate uptake by human Caco‐2 cell monolayers (Arvans et al., 2020). NHE3 is a well‐established target of cAMP, inhibited through changes in phosphorylation (Moe, 1999) and endocytosis to reduce sodium absorption with a parallel increase in anion secretion (Donowitz & Li, 2007; Xu et al., 2018), but the same cannot be said of Cl^−^/HCO_3_^−^ exchangers directly. For example, DRA appears to be retrieved from the apical membrane together with NHE3 (Musch et al., 2009) and PAT1 seems to be unaffected by elevated cAMP in the mouse small intestine (Walker et al., 2009; Wang et al., 2005). NHE3 has been shown to be physically coupled to bicarbonate chloride exchangers through PDZ domain containing proteins such as the various NHERF (NHE regulatory factor) proteins (Lamprecht et al., 2009; Lohi et al., 2003) which appear to be key for many of the joint functions of NHE3 and anion exchange and the regulation of electrolyte transport in general (Ghishan & Kiela, 2012; Kato & Romero, 2011; Seidler et al., 2009). Although, oxalate transport by the intestine involves Cl^−^/HCO_3_^−^ exchange pathways and appears to be associated with NaCl cotransport, it is not clear whether it is also a part of the coordinated function with NHE3.

To test the hypothesis that NHE3 might be involved in the regulation of oxalate transport through its interaction with Cl^−^/HCO_3_^−^ exchange, this study uses an NHE3 KO mouse model. Similar to the DRA KO mouse, which displays perturbed oxalate homeostasis (Freel et al., 2013), the previously established NHE3 KO mouse strain exhibits a persistent, lifelong diarrheal phenotype (Gawenis et al., 2002; Schultheis, Clarke, Meneton, Miller, et al., 1998). We assessed whether renal handling of oxalate was perturbed in NHE3 KO mice by measuring urinary oxalate outputs in vivo and intestinal oxalate transport rates in vitro, compared to WT counterparts. Intestinal oxalate and sodium transport were measured across the distal ileum, cecum, and distal colon using the Ussing chamber. Utilizing the NHE3 KO mouse allowed us to investigate the role of NHE3 in both the intestine and kidneys without concern about appropriate dosing levels and potential off‐target effects or incomplete inhibition which can be a problem when utilizing pharmacological inhibitors. With reliable antibodies available, immunohistochemistry was used to qualitatively assess NHE3 expression in these three intestinal segments. To assess a potential role of NHE3 in cAMP‐modulated oxalate transport, forskolin was applied to stimulate intracellular cAMP production and survey the effects on oxalate transport.

## 2 METHODS

### 2.1 Experimental animals

NHE3 KO mice and wild‐type (WT) litter mates on an FVB/N background were obtained from heterozygous breeding pairs housed in the University of Florida's Association for Assessment and Accreditation of Laboratory Animal Care‐accredited facility in the Biomedical Sciences Building. Mice were given free access to standard chow (Teklad diet 7919; Envigo) and sterile water. NHE3 KO mice were given Pedialyte® with drinking water (50% v/v) from weaning in an attempt to offset electrolyte and fluid losses associated with diarrhea and to enhance survival. WT mice were not given access to Pedialyte® from weaning as they do not display diarrhea, however, prior to experiments WT mice had Pedialyte® added to their drinking water (50% v/v) at least 3 days prior to the collection of urine, 1 day prior to collection of tissues for histology, and 2–21 days prior to the measurement of ion fluxes. A reviewer suggested that WT mice with induced diarrhea may provide an interesting comparison group to account for the effects of the diarrhea in the NHE3 KO mice; however, such a group was not used due to potential interactions between the mechanisms of diarrhea induction and oxalate transport mechanisms (Freel et al., 1998; Hatch, Freel, & Vaziri, 1993, 1994b). Mice used were between ages 59 and 276 days and weighed between 19 and 40.5 g. Generation of the NHE3 KO mouse model is described elsewhere (Schultheis, Clarke, Meneton, Harline, et al., 1998; Schultheis, Clarke, Meneton, Miller, et al., 1998) and were generously provided by Dr Bryan Mackenzie of the University of Cincinnati. All animal experimentation was approved by the University of Florida Institutional Animal Care and Use Committee (IACUC) and performed in accordance with the National Institutes of Health “Guide for the Care and Use of Laboratory Animals.”

### 2.2 Urine collection and oxalate analysis

NHE3 KO and WT mice were placed in individual metabolic cages for a 24‐h urine collection during which they had access to food and Pedialyte® with drinking water (50% v/v). Sodium azide (20 µl, 2% wt/vol) was used as a preservative in urine collection tubes with mineral oil (75 µl) to prevent evaporation. Following retrieval of urine, total collection volume was determined and 250 µl of aliquot was acidified with 3 N HCl to prevent oxalogenesis in preparation for determination of oxalate concentration using an enzyme‐based assay kit (Trinity Biotech, St Louis, MO). The remaining volume was diluted with ultra‐pure water and frozen at −20°C for later determination of creatinine and sodium concentrations.

### 2.3 Transepithelial flux experiments

Transepithelial, unidirectional fluxes of oxalate and sodium were measured across intact sections of the distal ileum, cecum, and distal colon under symmetrical short‐circuit conditions using Ussing chambers and radiolabeled ^14^C‐oxalate and ^22^Na^+^.

Distal ileum, cecum, and distal colon tissue sections were obtained from NHE3 KO (*n* = 7 female and *n* = 4 male) and WT (*n* = 8 female and *n* = 4 male) mice. Mice were killed with 100% CO_2_ and exsanguinated via cardiac puncture. Blood obtained from cardiac puncture was collected using heparinized syringes and centrifuged at a force of 800 × g for 20 min at 5°C. The resultant plasma was collected and stored at −20°C for later determination of creatinine concentration. Immediately after exsanguination, the intestines were removed and placed in ice‐cold flux buffer. Individual tissue sections (~ 2 cm long each) of the distal ileum (up to 4 cm proximally from the ileocecal valve), cecum, and distal colon (up to 4 cm proximally from the peritoneal border) were then prepared by cutting along the mesenteric line and mounting on Ussing chamber sliders (P2304; Physiologic Instruments, San Diego, CA, USA) to expose 0.3 cm^2^ of surface area. These intestinal segments were chosen and left intact to match the segments examined in previous studies that identified DRA and PAT1 as oxalate transporters (Freel et al., 2006, 2013; Whittamore & Hatch, 2020). Furthermore, sodium transport across the murine distal ileum, cecum, and distal colon has only been previously measured in intact tissues (Charney et al., 2004; Homaidan et al., 1999). Using intact tissue also lessened the risk of rendering tissues unusable through damage when stripping. Each slider was then mounted into Ussing chambers (P2300; Physiologic Instruments) and 4 ml of flux buffer was placed on each side of the tissue. Flux buffer (pH 7.4) in the chambers was maintained at 37°C and bubbled with a mixture of O_2_ (95%) and CO_2_ (5%). The flux buffer contained the following solutes (mM): 139.8 Na^+^, 118.2 Cl^−^, 25.0 HCO_3_^−^, 5.0 K^+^, 1.6 HPO_4_^2−^, 1.0 Ca^2+^, 1.0 Mg^2+^, 0.4 H_2_PO_4_^−^, 1.0 SO_4_^2−^, and 10 glucose. The potential difference across the mounted tissues was maintained at 0 mV using an automatic voltage clamp (VCCM6 or VCC600; Physiologic Instruments). Tetrodotoxin and indomethacin were not used in case the pathways they inhibit are integral to regulation of intestinal oxalate transport. Glucose was included in the mucosal buffer as the presence of glucose activates sodium glucose co‐transport pathways in the small intestine, a pathway which may also be regulated by NHE3 (Chan et al., 2018) and it is unknown whether the associations between NHE3 and this pathway could be involved in oxalate transport in the intestine.

Unidirectional fluxes of oxalate and sodium were measured by the addition of both 0.27 µCi of ^14^C‐oxalate and 0.09 µCi of ^22^Na^+^ to either the mucosal or serosal side of the tissue which was then designated as the “hot” side, with the opposing side designated as the “cold” side. Transepithelial current (I_sc_) and potential difference were recorded and samples of flux buffer (1 ml) were taken from the cold side at 15‐minute intervals for 1 h. Samples of flux buffer from the cold side were replaced immediately with 1 ml of pre‐warmed flux buffer. After an hour, intracellular cAMP was stimulated and maintained by a cocktail of forskolin (FSK) and 3‐isobutyl‐1‐methylxanthine (IBMX) added to both sides of the chambers to yield a final concentration of 10 µM and 100 µM of FSK and IBMX, respectively. Recordings and sampling from the cold side then continued at 15‐minute intervals for another hour. Samples of the hot side buffer (50 µl) were taken at the beginning and end of experiments to calculate specific activity. The activity of samples was determined using liquid scintillation detection (Beckman LS6500; Beckman Coulter, Fullerton, CA). Where necessary, buffer was added to samples to achieve a volume of 1 ml and then samples were mixed with 5 ml of scintillation cocktail (Ecoscint A; National Diagnostics, Atlanta, GA, USA). Liquid scintillation detection was then performed for both radiolabels simultaneously, and the results were quench corrected after accounting for overlap in the detection windows. The method for dual‐label detection and quench correction was verified using a series of external standards.

### 2.4 Sodium and creatinine measurements

Urine and plasma creatinine and sodium concentrations were measured on a Beckman Coulter AU5800 chemistry analyzer at UF Health Pathology Laboratories (Gainesville, FL). Sodium was measured using an ion selective electrode, whereas creatinine was determined using a kinetic modification of the Jaffé procedure in which creatinine reacts with picric acid. The rate of change in absorbance is proportional to the creatinine concentration in the sample. The creatinine calibrator is traceable to an isotope dilution mass spectrometry (IDMS) reference method used by the National Institutes of Standards and Technology (NIST).

### 2.5 Histology and immunohistochemistry

Distal ileum, cecum, and distal colon tissue sections were obtained from NHE3 KO (*n* = 2 female and *n* = 2 male) and WT (*n* = 1 female and *n* = 3 male) mice. Mice were killed with 100% CO_2_ and cervical dislocation. Intestines were then removed and placed immediately into ice‐cold phosphate‐buffered saline (PBS). The intestine was then divided into three sections for histology: the distal ileum (approximately up to 8 cm proximal to the ileocecal valve), the cecum, and the distal colon (up to 8 cm proximal to the peritoneal border). Each section was then placed on a moistened filter paper and opened along the mesenteric border. Tubular sections (i.e., distal ileum and colon) were then pinned flat on wax dissection blocks and rinsed with cold PBS to remove contents. The sections were then rolled onto toothpicks, proximal end first, mucosal side out, to create “Swiss rolls” as previously described (Moolenbeek & Ruitenberg, 1981).

“Swiss rolls” were created from the cecum as follows. The cecum, once opened along the mesenteric border, was laid flat and dragged mucosal side down on a PBS wetted filter paper to remove contents. The cecum was then laid mucosal side up and stretched out flat on a clean PBS wetted filter paper. Using a razor blade, a strip approximately 6 mm wide was cut along the central axis, from the apex ceci to the ampulla ceci, as defined previously (Snipes, 1981). These strips were then rinsed and rolled onto toothpicks similarly to the distal ileum and colon sections, but apex ceci end first.

Each “Swiss roll” was pinned through with a 27.5‐gauge needle, fixed in 10% neutral‐buffered formalin for 16–21 h at room temperature, and then embedded in paraffin for subsequent sectioning and staining at the University of Florida's Molecular Pathology core facility.

Antigen retrieval was performed using a citrate buffer. Nonspecific binding was blocked using 2.3% normal goat serum. Incubation with the primary antibody, a rabbit anti‐rat NHE3 polyclonal antibody (StressMarq Biosciences Cat# SPC‐400, RRID: AB_10643557, lot number 140501) diluted at 1:250, occurred for 60 minutes. Incubation with the secondary antibody, anti‐rabbit IgG HRP‐linked antibody, was for 30 min. Reactivity was visualized using the Vector DAB chromogen and a counterstained with hematoxylin/bluing from Biocare.

### 2.6 Calculations and statistics

Changes in activity of the cold side samples were used to calculate the unidirectional flux of oxalate and sodium. Unidirectional flux refers to the rate of movement from the mucosal (M) to serosal (S) side of the tissue ($J_{\mathrm{ms}}^{\mathrm{ion}}$) (i.e., the absorptive direction) and from the serosal to the mucosal side of the tissue ($J_{\mathrm{sm}}^{\mathrm{ion}}$) (i.e. the secretory direction). Net flux ($J_{\mathrm{net}}^{\mathrm{ion}}$) was calculated by $J_{\mathrm{net}}^{\mathrm{ion}}$ =$J_{\mathrm{ms}}^{\mathrm{ion}}$–$J_{\mathrm{sm}}^{\mathrm{ion}}$ from tissue pairs, so that a negative $J_{\mathrm{net}}^{\mathrm{ion}}$ value indicates a net secretory movement. Tissue pairs were assigned based on matching average tissue conductance (G_T_) in the first hour of the experiment as closely as possible. Tissues were only paired if the difference in G_T_ between them was <±25% for the large intestine and <±15% for the small intestine. G_T_ was calculated from I_sc_ and potential difference using Ohm's law.

Differences in urinary parameters and control period fluxes between NHE3 KO and WT mice were assessed for statistical significance using independent *t*‐tests unless otherwise stated. For data which failed the Shapiro–Wilk test for normality, the Mann–Whitney rank sum test was used in place of a *t*‐test. For data which failed the Brown–Forsythe test of equal variances, Welch's *t*‐test was used instead. Difference in average ion transport rates before and after the application of FSK/IBMX were assessed by a paired *t*‐test or a Wilcoxon signed‐rank test if the data failed the Shapiro–Wilk test for normality. These analyses were performed in SigmaPlot 14.

Results in the text are presented as the mean ±SEM (*n*) for either the first hour of the experiment or the second hour of the experiment, when FSK and IBMX were added. Significance was determined by *p* values <0.05.

## 3 RESULTS

### 3.1 Phenotype and metabolic characterization of NHE3 KO mice

In general, NHE3 KO mice were observed to have severe diarrhea with distended abdomens, resulting from the accumulation of large amounts of liquid and gas within the intestines, especially within the ceca. This was associated with a large increase in fluid consumption, nearly double that of WT mice (Table 1). Mice were not age matched due to small litter sizes and poor survival of NHE3 KO individuals, as such the KO mice used were approximately one third the age of their WT counterparts (Table 1). Body mass and food intake were not significantly different between groups, however we noted that the body composition of NHE3 KO mice was visibly different with a larger proportion of body mass likely to come from the intestinal contents. Once intestines were removed from NHE3 KO mice, other internal organs appeared smaller with less interstitial fat and muscle mass compared to WT mice, consistent with previous observations (Laubitz et al., 2008).

**TABLE 1:** Information on mice from 24‐h metabolic cage collections, averaged for WT and NHE3 KO mice

	WT	NHE3 KO	*p*
Mouse age (days)	184 ± 26 (9)	54 ± 1 (9)	0.004
Sex (F/M)	5/4	4/5	
Mouse weight (g)	28 ± 3 (9)	24 ± 1 (9)	0.452
Food intake (g/24 h)	2 ± 1 (9)	3 ± 1 (9)	0.131
Fluid intake (ml/24 h)	5.18 ± 0.99 (9)	10.48 ± 2.5 (9)	0.027
Urine volume (ml/24 h)	2.44 ± 0.6 (9)	2.51 ± 0.77 (9)	0.536
Urinary oxalate excretion rate (µmol/24 h)	0.763 ± 0.224 (9)	0.881 ± 0.22 (9)	0.860
Urinary creatinine excretion rate (µmol/24 h)	4.4 ± 0.6 (9)	2.8 ± 0.4 (8)	0.042^‡^
Urinary oxalate/creatinine excretion rate (Ox:Cre)	0.156 ± 0.023 (9)	0.333 ± 0.049 (8)	0.008^‡^
Urinary sodium excretion rates (µmol/24 h)	269 ± 32 (9)	53 ± 12 (8)	<0.001^†^
Plasma creatinine (µM)	12 ± 1 (9)	20 ± 1 (8)	<0.001^‡^
Plasma sodium (mM)	153 ± 1 (9)	150 ± 1 (8)	0.048^‡^
Creatinine clearance (µl/min)	271 ± 53 (9)	95 ± 12 (7)	0.003
Sodium clearance (µl/min)	1.22 ± 0.143 (9)	0.209 ± 0.046 (7)	<0.001^†^
Sodium clearance ratio	0.00491 ± 0.00042 (9)	0.00212 ± 0.00032 (7)	<0.001^‡^

Differences between WT and NHE3 KO mice were assessed using a Mann–Whitney rank sum test, a *t*‐test (indicated with ^‡^), or a Welch's *t*‐test in the case of unequal variances (indicated with^†^).

Urinary output volumes for NHE3 KO mice were not significantly different from WT mice (Table 1). There was no significant difference in urinary oxalate excretion rates observed, when expressed as µmol/24 h; however urinary oxalate excretion rates were approximately doubled in NHE3 KO mice when expressed as a ratio of creatinine. Plasma creatinine concentrations in NHE3 KO mice were nearly double than that of the WT mice, which was statistically significant (Table 1). The loss of NHE3 had a large impact on urinary creatinine and sodium excretion, creatinine and sodium clearance and the sodium clearance ratio, which were all significantly lower in NHE3 KO mice (Table 1).

### 3.2 Immunohistochemistry

**FIGURE 1:**
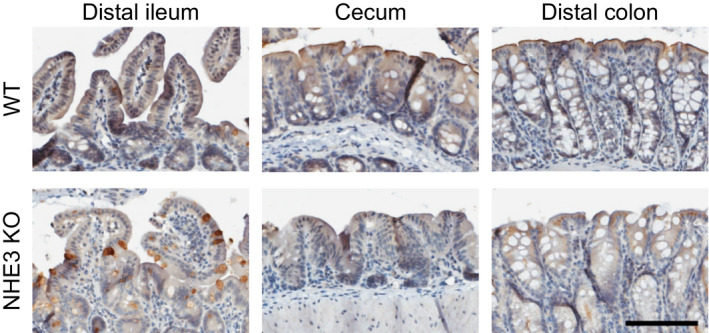
Immunohistochemical staining of NHE3 in different intestinal sections of wild‐type (WT) mice and negative controls from NHE3 KO mice. Black bar represents 100 µm

NHE3 expression was evident in the distal ileum, cecum, and distal colon (Figure 1). Localization of NHE3 expression was confined discreetly to the apical, luminal facing edges of surface cells in the large intestine with little evidence of expression in the crypts. Similarly, in the distal ileum, staining appeared stronger at the apical edge of the villi tips, but was less discreet and more inconsistent along the tissue sections examined when compared to the large intestine. Nonspecific intracellular binding was evident in the large intestine and in the goblet cells of the distal ileum of both the WT and NHE3 KO tissues. Despite this, there was no evidence of staining along the luminal facing edge of apical epithelial in KO tissues suggesting this is specific binding to NHE3 in the WT.

### 3.3 Transepithelial fluxes in the distal ileum

The distal ileum secreted oxalate and absorbed sodium on a net basis and the absence of NHE3 led to no statistically significant differences in transepithelial oxalate or sodium fluxes (Table 2). $J_{\mathrm{ms}}^{\mathrm{Na}}$ decreased by approximately 25% in NHE3 KO mice; however this was not statistically significant. The only statistically significant differences observed between WT and NHE3 KO were in I_sc_ and G_T_ which were both lower in NHE3 KO distal ileum. The WT sustained an average I_sc_ of −11.83 ± 1.08 (16) µeq/cm^2^·h, whereas the average I_sc_ for the NHE3 KO was −8.16 ± 1.38 (16) µeq/cm^2^·h. Accordingly, G_T_ was 30.71 ± 3.75 (16) mS/cm^2^ in KO mice compared to 43.55 ± 2.51 (16)) mS/cm^2^ in the WT.

**TABLE 2:** Comparison of the mean ±SEM (*n*) intestinal oxalate and sodium flux measurements for the distal ileum in WT and NHE3 KO mice under basal symmetrical, short‐circuit conditions in vitro

Genotype	Oxalate flux (pmol/cm^2^·h)			Sodium flux (µmol/cm^2^·h)			Electrophysiology	
	M to S	S to M	Net	M to S	S to M	Net	I_sc_ (µeq/cm^2^·h)	G_T_ (mS/cm^2^)
WT	27.86 ± 8.07 (8)	42.42 ± 6.27 (8)	−14.56 ± 8.46 (8)	21.39 ± 3.07 (8)	14.18 ± 1.66 (8)	7.22 ± 4.32 (8)	−11.83 ± 1.08 (16)	43.55 ± 2.51 (16)
NHE3 KO	22.30 ± 2.90 (8)	47.70 ± 9.07 (8)	−25.40 ± 9.52 (8)	16.73 ± 2.86 (8)	11.91 ± 1.68 (8)	4.81 ± 1.74 (8)	−8.16 ± 1.38 (16)	30.71 ± 3.75 (16)
	0.959^†^	0.639	0.409	0.285	0.345	0.618	0.045*	0.012*^,†^

“M to S” indicates measurements taken in the mucosal to serosal direction, “S to M” indicates measurements in the serosal to mucosal direction, and “Net” is the “S to M” subtracted from the “M to S” measurements in tissues paired based on conductance (G_T_). Transepithelial current is indicated by “Isc.” Below each variable measured a *p* value from a *t*‐test unless data failed Shapiro–Wilk test of normality, in which case a Mann–Whitney rank sum test was used and is indicated by ^†^. Values considered significant (i.e., *p* < 0.05) are highlighted with *.

**FIGURE 2:**
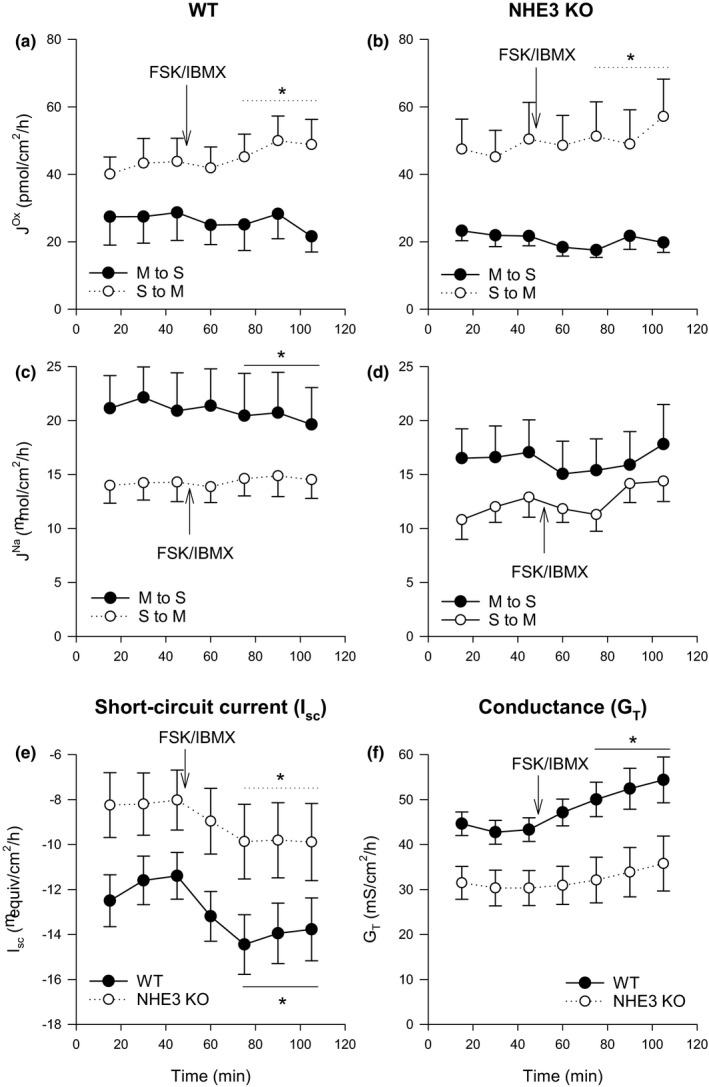
The effect of a cocktail of 10 µM and 100 µM of FSK and IBMX, respectively, on transepithelial fluxes of oxalate $J^{\mathrm{ox}}$ and sodium $J^{\mathrm{Na}}$ in wild‐type (WT) and NHE3‐knockout (NHE3 KO) murine distal ileum. Arrows labelled FSK/IBMX indicate the time point at which the cocktail was added. Unidirectional transepithelial fluxes of oxalate (a‐b) and sodium (c‐d) were measured simultaneously with each point representing the mean from *n* = 7 to 8 epithelial sections from WT or NHE3 KO mice. Short‐circuit current (I_sc_) and transepithelial conductance (G_T_) responses for WT and NHE3 KO ceca are shown in (e) and (f) with each point representing *n* = 16 epithelial sections from WT or NHE3 KO mice. Error bars show standard error of the mean. S to M indicates serosal to mucosal direction of flux and M to S mucosal to serosal. Average measurements that were statistically significantly different (*p* < 0.05) after the addition of FSK/IBMX as determined using a paired *t*‐test (or a signed‐rank test if more appropriate) are indicated by *

Bilateral application of FSK/IBMX to WT distal ileum significantly increased net oxalate secretion from an average of −14.56 ± 8.46 (8) to −24.31 ± 8.46 (8) pmol/cm^2^·h, through a small but significant increase in $J_{\mathrm{sm}}^{\mathrm{ox}}$ (Figure 2a). $J_{\mathrm{ms}}^{\mathrm{ox}}$ was also lower but not significantly so (*p* = 0.138, paired *t*‐test). In the NHE3 KO distal ileum, there was a similar stimulation of net oxalate secretion by FSK/IBMX (from −25.40 ± 9.52 (8) pmol/cm^2^·h to −32.82 ± 10.85 (8) pmol/cm^2^·h, *p* = 0.012, paired *t*‐test) through a similar modest increase in $J_{\mathrm{sm}}^{\mathrm{ox}}$.

FSK/IBMX resulted in a very modest (<10%), but statistically significant decrease in the absorptive sodium flux of WT distal ileum (Figure 2c). Correspondingly, net sodium absorption was reduced to an average of 4.79 ± 4.72 (8) µmol/cm^2^·h after FSK/IBMX application (*p* = 0.013, paired *t*‐test). In the NHE3 KO distal ileum, there were no statistically significant changes to $J_{\mathrm{ms}}^{\mathrm{Na}}$ or $J_{\mathrm{sm}}^{\mathrm{Na}}$ after application of FSK/IBMX application (*p* = 0.485, Figure 2d) and net sodium absorption was not significantly changed (*p* = 0.110).

FSK/IBMX induced a statistically significant increase in I_sc_ in both the WT and the NHE3 KO distal ileum (Figure 2e) and a slight increase in G_T_ which displayed a similar trend over time (Figure 2f), but was slightly more pronounced and statistically significant in the WT only.

### 3.4 Transepithelial fluxes in the cecum

In general, unidirectional fluxes of oxalate were lower in the NHE3 KO mouse cecum compared to the WT (Table 3), however, on average, only $J_{\mathrm{sm}}^{\mathrm{ox}}$ was statistically significantly lower compared to WT (Table 3). Neither WT nor NHE3 KO ceca supported a net oxalate flux under basal conditions (Table 3). Basal unidirectional sodium fluxes were also lower in the NHE3 KO cecum relative to WT (*p* < 0.001 for both $J_{\mathrm{ms}}^{\mathrm{Na}}$ and, $J_{\mathrm{sm}}^{\mathrm{Na}}$ *t*‐test) (Table 3), and again, net movement of sodium was negligible for both the WT and KO. Corresponding to the lower unidirectional fluxes of oxalate and sodium, I_sc_ and G_T_ were also statistically significantly lower in the NHE3 KO compared to the WT cecum (Table 3).

**TABLE 3:** Comparison of the mean ±SEM (*n*) intestinal oxalate and sodium flux measurements for the cecum in WT and NHE3 KO mice under basal symmetrical, short‐circuit conditions in vitro

Genotype	Oxalate flux (pmol/cm^2^·h)			Sodium flux (µmol/cm^2^·h)			Electrophysiology	
	M to S	S to M	Net	M to S	S to M	Net	I_sc_ (µeq/cm^2^·h)	G_T_ (mS/cm^2^)
WT	13.46 ± 3.44 (11)	18.19 ± 2.19 (11)	−4.73 ± 4.00 (11)	10.26 ± 0.78 (11)	9.25 ± 0.73 (11)	1.01 ± 0.99 (11)	−3.41 ± 0.26 (22)	16.42 ± 0.56 (22)
NHE3 KO	10.11 ± 2.69 (11)	13.77 ± 0.78 (11)	−3.18 ± 2.05 (11)	5.01 ± 0.35 (11)	4.77 ± 0.22 (11)	0.24 ± 0.25 (11)	−1.84 ± 0.17 (22)	8.58 ± 0.34 (22)
	0.088^†^	0.018*^,†^	0.793^†^	<0.001*	<0.001*^,†^	0.466^‡^	<0.001*	<0.001*

“M to S” indicates measurements taken in the mucosal to serosal direction, “S to M” indicates measurements in the serosal to mucosal direction, and “Net” is the “S to M” subtracted from the “M to S” measurements in tissues paired based on conductance (G_T_). Transepithelial current is indicated by “Isc.” Below each variable measured a *p* value from a *t*‐test unless data failed Shapiro–Wilk test of normality, in which case a Mann–Whitney rank sum test was used and is indicated by ^†^. In cases where the assumption of equal variances was violated (marked with ^‡^), Welch's *t*‐test was used. Values considered significant (i.e., *p* < 0.05) are highlighted with *.

**FIGURE 3:**
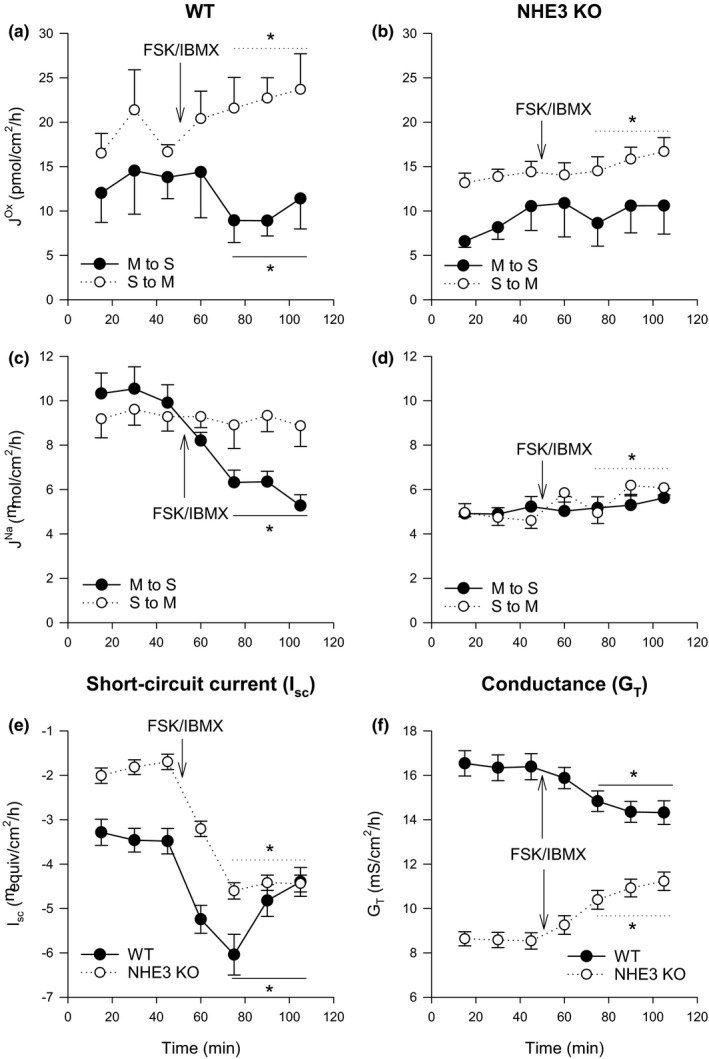
The effect of a cocktail of 10 µM and 100 µM of FSK and IBMX, respectively, on transepithelial fluxes of oxalate $J^{\mathrm{ox}}$ and sodium $J^{\mathrm{Na}}$ in wild‐type (WT) and NHE3‐knockout (NHE3 KO) murine cecum. Arrows labelled FSK/IBMX indicate the time point at which the cocktail was added. Unidirectional transepithelial fluxes of oxalate (a‐b) and sodium (c‐d) were measured simultaneously with each point representing the mean from *n* = 11 epithelial sections from WT mice and *n* = 9 to 11 epithelial sections from NHE3 KO mice. Short‐circuit current (_Isc_) and transepithelial conductance (G_T_) responses for WT and NHE3 KO ceca are shown in (e) and (f) with each point representing *n* = 22 epithelial sections from WT or NHE3 KO mice. Error bars show standard error of the mean. S to M indicates serosal to mucosal direction of flux and M to S mucosal to serosal. Average measurements that were statistically significantly different (*p* < 0.05) after the addition of FSK/IBMX as determined by a paired *t*‐test (or a signed‐rank test if more appropriate) are indicated by *

The application of FSK/IBMX induced robust net oxalate secretion in the WT cecum (Figure 3a) to −12.99 ± 3.85 (11) pmol/cm^2^·h (*p* = 0.002, paired *t*‐test) after stimulation in WT tissues, representing an average 2.8 fold increase, achieved by a sharp reduction in $J_{\mathrm{ms}}^{\mathrm{ox}}$, and steady increase in $J_{\mathrm{sm}}^{\mathrm{ox}}$ (Figure 3a).

In the NHE3 KO cecum there was a smaller, statistically significant increase in $J_{\mathrm{sm}}^{\mathrm{ox}}$ after FSK/IBMX application (*p* = 0.036) corresponding to an overall increase with time. However, the significant drop in $J_{\mathrm{ms}}^{\mathrm{ox}}$ after FSK/IBMX application was absent in the NHE3 KO cecum (*p* = 0.577) and, correspondingly, net transport was not statistically significantly different (Figure 3b).

$J_{\mathrm{ms}}^{\mathrm{Na}}$ was significantly reduced by FSK/IBMX in the WT cecum by ~40% to 5.99 ± 0.34 (11) µmol/cm^2^·h (*p* < 0.001, paired *t*‐test) but there was no change in response to FSK/IBMX in the NHE3 KO (*p* = 0.119 paired *t*‐test) (Figure 3).

In the WT cecum, the decrease in $J_{\mathrm{ms}}^{\mathrm{Na}}$ in response to FSK/IBMX led to a small secretory net flux of −3.00 ± 0.79 (11) µmol/cm^2^·h (*p* < 0.001, paired *t*‐test). In the NHE3 KO cecum net sodium transport was negligible both before and after FSK/IBMX application (Figure 3).

Despite the inability to alter sodium transport by FSK/IBMX application in the NHE3 KO ceca, I_sc_ still increased in both the WT and KO indicative of net anion (Cl^−^ and/or HCO_3_^−^) secretion (Figure 3e). The magnitude of the change was similar between WT and KO cecum. As seen in the distal ileum, G_T_ was lower in the NHE3 KO and, interestingly, the application of FSK/IBMX decreased G_T_ in the WT, whereas in the KO conductance increased (Figure 3).

### 3.5 Transepithelial fluxes in the distal colon

Oxalate transport rates were similar in both the WT and NHE3 KO distal colon, each supporting a moderate net secretion with no statistically significant differences between the two groups (Table 4). The WT displayed a very small net sodium absorption. Unidirectional sodium fluxes were slightly lower in the NHE3 KO distal colon, but the differences were not statistically significant and correspondingly net sodium flux was also similar (Table 4).

**TABLE 4:** Comparison of the mean ±SEM (*n*) intestinal oxalate and sodium flux measurements for the distal colon in WT and NHE3 KO mice under basal symmetrical, short‐circuit conditions in vitro

Genotype	Oxalate flux (pmol/cm^2^·h)			Sodium flux (µmol/cm^2^·h)			Electrophysiology	
	M to S	S to M	Net	M to S	S to M	Net	I_sc_ (µeq/cm^2^·h)	G_T_ (mS/cm^2^)
WT	14.06 ± 2.88 (10)	27.74 ± 4.6 (10)	−13.67 ± 5.38 (10)	9.57 ± 0.83 (10)	6.83 ± 1.26 (10)	2.74 ± 1.58 (10)	−3.52 ± 0.29 (20)	12.00 ± 1.08 (20)
NHE3 KO	18.14 ± 3.09 (9)	31.29 ± 6.32 (9)	−13.15 ± 6.01 (9)	7.10 ± 1.03 (9)	5.98 ± 0.65 (9)	1.12 ± 0.65 (9)	−3.82 ± 0.72 (18)	15.45 ± 1.35 (18)
	0.347	0.633	0.949	0.075	0.903^†^	0.094^†^	0.715^†^	0.037*^,†^

“M to S” indicates measurements taken in the mucosal to serosal direction, “S to M” indicates measurements in the serosal to mucosal direction, and “Net” is the “S to M” subtracted from the “M to S” measurements in tissues paired based on conductance (G_T_). Transepithelial current is indicated by “Isc.” Below each variable measured a *p* value from a *t*‐test unless data failed Shapiro–Wilk test of normality, in which case a Mann–Whitney rank sum test was used and is indicated by ^†^. Values considered significant (i.e., *p* < 0.05) are highlighted with *.

Unlike the NHE3‐KO distal ileum and cecum, G_T_ was statistically significantly higher with the absence of NHE3 from the distal colon (Table 4). Also unique to the distal colon was a significantly higher I_sc_ (Table 4), whereas in the NHE3 KO distal ileum and cecum, there was a statistically significant decrease.

**FIGURE 4:**
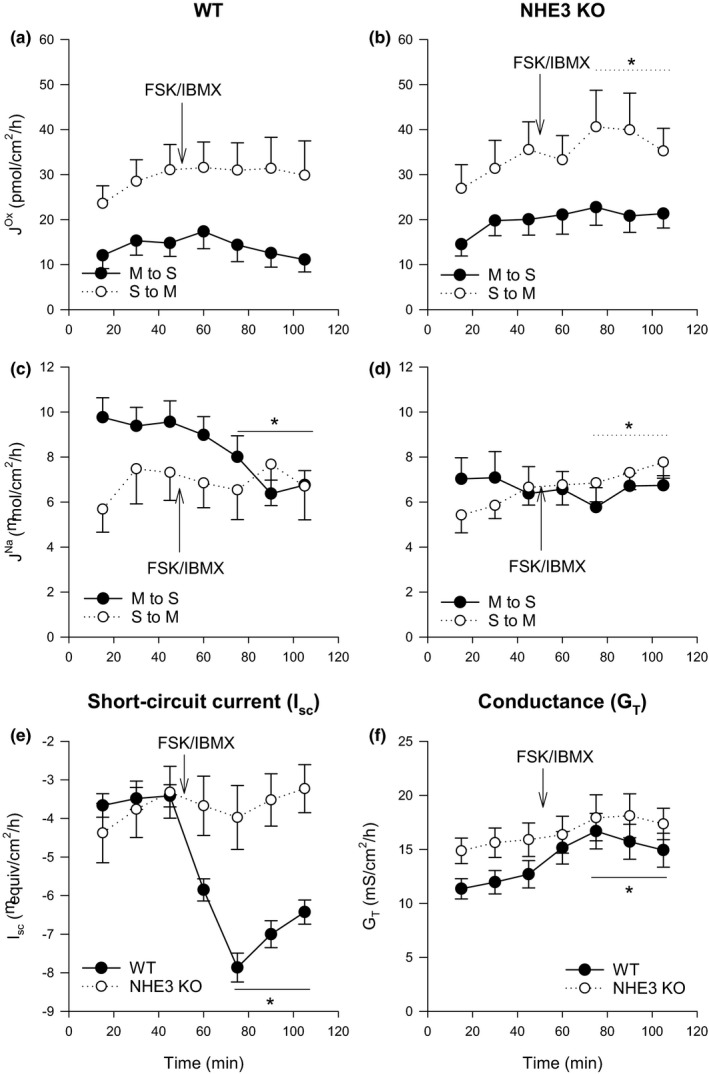
The effect of a cocktail of 10 µM and 100 µM of FSK and IBMX, respectively, on transepithelial fluxes of oxalate $J^{\mathrm{ox}}$ and sodium $J^{\mathrm{Na}}$ in wild‐type (WT) and NHE3‐knockout (NHE3 KO) murine distal colon. Arrows labelled FSK/IBMX indicate the time point at which the cocktail was added. Unidirectional transepithelial fluxes of oxalate (a‐b) and sodium (c‐d) were measured simultaneously with each point representing the mean from *n* = 10 epithelial sections from WT or NHE3 KO mice. Short‐circuit current (I_sc_) and transepithelial conductance (G_T_) responses for WT and NHE3 KO ceca are shown in (e) and (f) with each point representing *n* = 20 epithelial sections from WT or NHE3 KO mice. Error bars show standard error of the mean. S to M indicates serosal to mucosal direction of flux and M to S mucosal to serosal. Average measurements that were statistically significantly different (*p* < 0.05) after the addition of FSK/IBMX as determined by a paired *t*‐test (or a signed‐rank test if more appropriate) are indicated by *

In the WT distal colon, the application of FSK/IBMX had no statistically significant stimulatory effect on oxalate fluxes (Figure 4a). In the NHE3 KO, FSK/IBMX led to a statistically significant 29% increase in $J_{\mathrm{sm}}^{\mathrm{ox}}$ but this did not result in any increase in the overall net secretion (*p* = 0.200, paired *t*‐test) (Figure 4b).

The application of FSK/IBMX to the WT distal colon led to a significant decrease in the absorptive sodium flux with $J_{\mathrm{ms}}^{\mathrm{Na}}$ (Figure 4c) decreasing on average to 7.05 ± 0.52 (10) µmol/cm^2^·h (*p* < 0.001, paired *t*‐test), enough to abolish net sodium absorption (*p* = 0.001, paired *t*‐test). Net sodium absorption was also abolished by FSK/IBMX in the NHE3 KO distal colon (*p* = 0.023, paired *t*‐test). However, unlike WT distal colon, $J_{\mathrm{ms}}^{\mathrm{Na}}$ was not significantly reduced and the small change in net sodium transport appeared to occur due to a continuous rise in $J_{\mathrm{sm}}^{\mathrm{Na}}$ throughout the experiment (Figure 4d).

In the WT distal colon FSK/IBMX application led to a statistically significant increase in I_sc_ as expected, from −3.52 ± 0.29 (20) to −7.1 ± 0.33 (20) µeq/cm^2^·h (*p* < 0.001, paired *t*‐test), however, in the NHE3 KO distal colon I_sc_ was unchanged (*p* = 0.269, paired *t*‐test). FSK/IBMX application increased G_T_ in both the WT and NHE3 KO distal colon in a similar fashion (Figure 4f), however, the increase was only statistically significant in the WT (*p* < 0.001 in the WT, Wilcoxon signed‐rank test and *p* = 0.061 in the NHE3 KO, paired *t*‐test).

## 4 DISCUSSION

Electroneutral sodium chloride and fluid absorption by the mammalian intestine is mediated by coupled Na^+^/H^+^ and Cl^−^/HCO_3_^−^ exchange located in the apical membrane of enterocytes. NHE3 has been identified as the main Na^+^/H^+^ exchanger and is highly regulated (Donowitz & Li, 2007; Xu et al., 2018). It is known that oxalate is transported by the accompanying chloride/bicarbonate exchangers, PAT1 (Freel et al., 2006) and DRA (Freel et al., 2013), that are functionally and physically associated with NHE3 (Musch et al., 2009; Seidler & Nikolovska, 2019; Seidler et al., 2008; Singh et al., 2010; Walker et al., 2008; Whittamore et al., 2013; Xia et al., 2014; Xu et al., 2018). Furthermore, the secondary messenger cAMP, which acts as an inhibitor of NHE3, can stimulate oxalate secretion in rabbit intestine (Freel et al., 1998; Hatch, Freel, & Vaziri, 1993, 1994b). Epinephrine, a stimulator of sodium absorption by NHE3 (Donowitz & Li, 2007), simultaneously enhances the absorptive flux of oxalate by the rabbit proximal colon with both sodium and oxalate absorption sensitive to the selective Na^+^/H^+^ exchange inhibitor, dimethyl amiloride (Hatch et al., 1993). With apparent links between NHE3 activity and oxalate absorption, the goal of this study was to assess whether NHE3 participates in oxalate homeostasis and the regulation of intestinal oxalate transport. This study suggests NHE3 likely plays a role in renal oxalate handling, whereas in the intestine, there was no evidence that NHE3 plays a role in basal oxalate transport in any segment examined but it was important for cAMP stimulation of oxalate secretion in the cecum only. NHE3 also contributed to net sodium absorption by the distal colon, and its inhibition by cAMP in the cecum and distal colon. As such, this study has revealed heterogeneity across the mouse intestine in terms of ion transport mechanisms. The segmental differences for both the role of cAMP and NHE3 in oxalate and sodium transport are discussed below.

### 4.1 The NHE3 KO phenotype and renal handling of oxalate

Observations of the NHE3 KO phenotype were generally similar to what has previously been reported in terms of intestinal distension, watery contents and reduced viscera (Laubitz et al., 2008; Schultheis, Clarke, Meneton, Miller, et al., 1998). The cohorts used in this study were approximately one third the age of their WT counterparts due to poor survivability of NHE3 KO mice, despite providing Pedialyte®. Poor survivability is a limitation of working with this KO model as noted previously (Liu et al., 2020) and the decision was made to conduct the experiments without age‐matched cohorts due to uncertainty over the ability and timescale to generate sufficient numbers again and because we have not previously seen any large correlations between age and intestinal oxalate fluxes, I_sc_ or G_T_ when examining results from WT mice between 62 and 318 days old (unpublished data). Despite the age difference, body mass was not different between WT and NHE3 KO mice (Table 1). However, we are aware that the body composition of these two genotypes of mice was different, with the intestinal contents likely contributing to a larger proportion of overall body mass in the NHE3 KO mice. Laubitz et al., (2008) showed their age‐matched NHE3 KO mice were smaller than WT mice; however, their mice were on a different genetic background (black Swiss), exhibited only “mild” diarrhea and did not require fluid and electrolyte supplementation to enable survival past weaning. Recently, an inducible intestine‐only NHE3 KO was developed which led to a transient decrease in body mass followed by an increase relative to non‐induced controls with NHE3 KOs eventually becoming heavier than their WT age‐matched counterparts (Xue et al., 2020). From previous studies, the intestinal tract accounts for approximately 9–15% of the body mass in the adult NHE3 KO compared to 6–7% of total body mass in WT mice (Schultheis, Clarke, Meneton, Miller, et al., 1998; Xue et al., 2020), and likely explains why younger NHE3 KO mice in the current study had a similar body mass to their older WT counterparts. This could have implications for interpretation of urinary oxalate and creatinine excretion rates as they are both closely linked to metabolic rate with the latter being tied to muscle mass (Perrone et al., 1992; Wyss & Kaddurah‐Daouk, 2000) which was visibly lower in the NHE3 KO cohort. Unfortunately, we did not weigh the carcass after removal of the intestine to confirm this, but creatinine excretion rates were approximately 65% of what was observed in WT mice (Table 1). It is important to consider that creatinine excretion is both a function of metabolic production and the ability of the kidneys to excrete it. Previous studies observed (GFR) in NHE3 KO mice to be 65 to 83% of what is observed in their WT counterparts on a “normal” diet (Ledoussal et al., 2001; Lorenz et al., 1999) and, when on a salt restricted diet with transgenic rescue of small intestinal defects, this can be as little as 28% of the WT (Woo et al., 2003). It is thought that GFR is lower in the absence of renal NHE3 to compensate for impaired fluid reabsorption by the proximal tubule rather than systemic hypovolemia (Woo et al., 2003). Unfortunately, it is difficult to assess how much of the reduction in NHE3 KO urinary creatinine excretion is due to decreased GFR and how much is the result of smaller muscle mass. However, despite this, plasma creatinine levels were almost double in the NHE3 KO mice, suggesting there may be some impairment of renal function. Correspondingly, creatinine clearance rates in the NHE3 KO mice were ~35% of those seen in the WT (Table 1). Furthermore, low muscle mass can lead to an overestimation of GFR when using creatinine clearance as a proxy due to the reduced metabolic supply of creatinine (Perrone et al., 1992). As such, it seems likely that reduced GFR has played a large role in reducing creatinine excretion in NHE3 KO mice.

Urinary oxalate excretion was not significantly different between WT and NHE3 KO mice when expressed as µmol/24 hours. Since oxalate is considered to be freely filtered by the glomerulus (Cattell et al., 1962; Greger et al., 1978), it is somewhat surprising that oxalate excretion expressed as µmol/day were not correspondingly reduced alongside creatinine clearance and presumably GFR. Reductions in estimated GFR in human patients leads to increases in plasma oxalate levels associated with lower 24 h urinary oxalate excretion (Perinpam et al., 2017). When oxalate excretion was expressed as a ratio of creatinine they were approximately doubled in NHE3 KO mice (Table 1). This might suggest significant changes to renal oxalate handling. Unfortunately, we did not measure plasma oxalate in order to calculate oxalate clearance due to the relatively large volume of plasma required for the enzyme‐based assay (Hatch et al., 1977), and the limited number of available donor KO mice. If we estimate plasma oxalate levels were approximately 20 µM, in agreement with previous measures from this lab (Freel et al., 2006, 2013; Hatch et al., 2011; Whittamore et al., 2018) for both WT and NHE3 KO mice, the oxalate:creatinine clearance ratio would be 0.09 for WT mice but more than three times higher for NHE3 KO mice at 0.31, indicative of a reduction in overall net reabsorption. This would indicate either increased secretion and/or decreased reabsorption by the nephron. In mice, oxalate secretion occurs in the proximal tubule via PAT1 (Knauf et al., 2019), and deletion of PAT1 has been shown to downregulate NHE3 activity in the proximal tubule (Petrovic et al., 2008) which might suggest a reciprocal decrease in PAT‐1‐mediated oxalate secretion following the loss of NHE3, however, this is not consistent with the apparent notion of increased oxalate secretion. These inferences assume that plasma oxalate levels were the same between WT and NHE3 KO mice, but we cannot discount that plasma oxalate levels may be higher in the NHE3 KO mice since GFR was likely impaired. In rat models of chronic renal failure (Hatch & Freel, 2003a; Hatch et al., 1994a), it would appear that there exists a remarkable ability to maintain urinary oxalate excretion rates. As such, in the current study the maintenance of urinary oxalate excretion, despite likely lower GFR may not directly be related to the absence of NHE3, rather the result of other innate systems for dealing with impaired kidney function such as those regulated by the renin–angiotensin–aldosterone system (RAAS) which is clearly activated in NHE3 KO mice as they exhibit increased serum aldosterone (Schultheis, Clarke, Meneton, Miller, et al., 1998). Overall, it would appear the renal handling of oxalate is disturbed in the NHE3 KO mouse, determining the exact mechanism responsible, however, requires further investigation.

### 4.2 The distal ileum

In the distal ileum, NHE3 appears to play a modest role, if any, in basal oxalate and sodium fluxes (Table 2) despite evidence of expression in this location (Figure 1) and previous evidence of coupled Na^+^/H^+^ and Cl^−^/HCO_3_^−^ exchange in the mammalian small intestine, including the ileum (Knickelbein et al., 1985; Turnberg et al., 1970; Walker et al., 2008; Xia et al., 2014). There were no statistically significant differences between WT and NHE3 KO mice in terms of either basal sodium or oxalate fluxes. The only statistically significant differences were I_sc_ and G_T_. Furthermore, the WT and NHE3 KO distal ileum appeared to share similar responses to FSK/IBMX application (Figure 2) inducing an increase in net oxalate secretion, I_sc_ and G_T_ and a decrease in net sodium absorption in both the NHE3 KO and WT mice.

In the mouse distal ileum, oxalate transport has been shown to occur through the bicarbonate chloride exchangers, PAT1 (Slc26a6) and DRA (Slc26a3), contributing to secretion and absorption, respectively (Freel et al., 2006, 2013). At least in other parts of the small intestine, PAT1 and DRA are known to be functionally and physically associated with NHE3 (Musch et al., 2009; Seidler & Nikolovska, 2019; Seidler et al., 2008; Singh et al., 2010; Walker et al., 2008; Xu et al., 2018); however, this study suggests NHE3 is not integral to the oxalate transport mechanism in murine distal ileum and basal oxalate fluxes were not dissimilar to what has previously been reported (Whittamore et al., 2015, 2018; Whittamore & Hatch, 2019). This occurred in the context of reduced G_T_ in the NHE3 KO distal ileum. Reduced G_T_ is consistent with previous Ussing chamber studies of NHE3 KO jejuna (Gawenis et al., 2002; Seidler et al., 2008), duodenum (Pan et al., 2012), and cecum (Rievaj et al., 2013) and consistent with evidence of NHE3 being directly involved in the regulation of tight junction permeability in the intestine (King et al., 2018; Turner et al., 2000). Since a reduction in transepithelial conductance would have brought about corresponding changes in paracellular oxalate flux, this may have masked any changes in transcellular flux resulting from interactions of NHE3 with PAT1 or DRA, making it difficult to definitively conclude that there is no role for NHE3 in basal oxalate transport by the murine distal ileum.

After the stimulation of cAMP, there was a modest but statistically significant increase in the secretory flux of oxalate in the WT distal ileum (Figure 2a); this was coincident with an increase G_T_ (Figure 2f), although we would have also expected an increase to the absorptive oxalate flux if this was exclusively due to increased paracellular permeability. cAMP levels have been shown to stimulate net oxalate secretion by the rabbit ileum (Freel et al., 1998) but not murine ileum (Freel et al., 2006), albeit in a different strain of mouse (C57BL/6) than used here (FVB/N). Differences in Ca^2+^ and cAMP‐stimulated intestinal ion secretion have been observed between different strains of mice (Flores et al., 2010). A similar increase in oxalate secretion was also observed in the NHE3 KO distal ileum in the current study (Figure 2b), suggesting that NHE3 is not an integral component of this mechanism.

Under basal conditions there was net sodium absorption by WT distal ileum (Table 2) similar to what has been observed previously (Charney et al., 2004). The NHE3 KO distal ileum also supported basal net sodium absorption, which was slightly lower compared to the WT but not significantly so (Table 2), suggesting NHE3 may not contribute to a large amount of ileal sodium absorption in the present experimental conditions. There was a statistically significant, but small, decrease in unidirectional and net sodium absorption after addition of FSK/IBMX in the WT distal ileum when tested by a paired *t*‐test (Figure 2c). In the NHE3 KO distal ileum, net sodium absorption decreased on FSK/IBMX addition more distinctly; however, the changes in the unidirectional sodium fluxes were transient and had recovered by the end of the experimental period (Figure 2d). As such, there is little evidence that NHE3 is required for cAMP control of sodium transport in the murine ileum, at least under the experimental conditions imposed here. In isolated murine ileum cells, NHE3 activity can be inhibited by cAMP (Murtazina et al., 2007; 2011). However, previous studies in the murine jejuna reached disparate conclusions as to whether NHE3 is necessary for cAMP inhibition of sodium absorption, finding that either NHE3 was dispensable for the actions of cAMP on sodium transport and short‐circuit current (Gawenis et al., 2002) or required for cAMP inhibition of sodium absorption (Seidler et al., 2008).

In the current study, the true role of NHE3 in the murine distal ileum could have been masked by sodium glucose co‐transport since the mucosal buffer also contained glucose. This would have supported the electrogenic sodium glucose co‐transporter, SGLT1 (Slc5a1) (Chen et al., 1995; Parent et al., 1992) representing another major route of sodium absorption in the small intestine (Kiela & Ghishan, 2016). A large amount of sodium‐driven glucose absorption could be obscuring changes to NHE3‐mediated Na^+^ transport. Previous studies on the NHE3 KO small intestine excluded glucose from the mucosal buffer and found unidirectional and net sodium absorption was statistically significantly lower in KO murine jejuna (Gawenis et al., 2002; Seidler et al., 2008) indicating a significant contribution to sodium absorption. In one of these studies (Seidler et al., 2008), glucose was added at the end of the experiment, after FSK, which caused unidirectional and net sodium absorption to increase (although not statistically significantly), and become more variable. The presence of glucose in the mucosal buffer could also explain the larger variability observed in $J_{\mathrm{ms}}^{\mathrm{Na}}$ compared to $J_{\mathrm{sm}}^{\mathrm{Na}}$ in the current study making it difficult to detect a reduction in sodium absorption. As such, it cannot be concluded with certainty that NHE3 is involved in sodium transport by the murine distal ileum.

Despite unclear results for sodium transport, basal transepithelial current was distinctly lower in the NHE3 KO distal ileum, both before and after FSK/IBMX addition (Figure 2e). As transport through NHE3 is electroneutral, this implies its absence may be regulating other (electrogenic) transport. Knockdown of NHE3 has previously been demonstrated to reduce sodium linked glucose absorption (Chan et al., 2018), which is electrogenic. In the current study, mean net sodium absorption was lower by 2.41 µmol/cm^2^·h in the NHE3 KO distal ileum, a magnitude that is not dissimilar to the magnitude of the difference in I_sc_ (3.67 µeq/cm^2^·h), although the change in net sodium transport was not statistically significant (Table 2).

Overall, cAMP can promote net oxalate secretion in both the WT and NHE3 KO distal ileum of the FVB/N mouse but with no evidence of involvement of NHE3 in either basal or cAMP stimulated oxalate transport. There were limitations in the ability of this study to detect the role of NHE3 in murine distal ileum sodium transport due to the concomitant activity of sodium‐glucose co‐transport, however, NHE3 appears to be important for basal rates of electrogenic ion transport in the distal ileum possibly through its interaction with SGLT1.

### 4.3 The cecum

NHE3 appears to play an important role in cAMP‐controlled changes in oxalate and sodium transport in the murine cecum. Basal rates of sodium and oxalate transport were generally lower in both directions across the NHE3 KO cecum; however there was negligible net transport of either of these ions in the WT and the NHE3 KO under these conditions (Table 3) and the lower unidirectional fluxes may be related to reduced paracellular permeability (as indexed by G_T_). However, the application of FSK/IBMX to raise intracellular levels of cAMP led to clear changes in the WT cecum in terms of oxalate and sodium transport but these changes were absent or muted in the NHE3 KO cecum suggesting possible involvement of NHE3 (Figure 3).

Unlike the distal ileum, PAT1 does not contribute to the secretory flux of oxalate in murine cecum, at least under basal conditions (Whittamore & Hatch, 2020). However, DRA has been shown to mediate the oxalate absorptive flux in this segment (Freel et al., 2013). At least in other parts of the intestine DRA is known to be functionally and physically associated with NHE3 (Ursula Musch et al., 2009; Seidler & Nikolovska, 2019; Seidler et al., 2008; Singh et al., 2010; Walker et al., 2008; Xia et al., 2014; Xu et al., 2018) but the expression of NHE3 protein was reportedly very low in rodent cecum (Talbot & Lytle, 2010). The expression of NHE3 mRNA has been shown in the murine cecum (Rievaj et al., 2013) and the ceca of NHE3 KO mice do have an altered luminal environment and impaired calcium absorption compared to WT mice (Engevik et al., 2013; Rievaj et al., 2013). In this study, NHE3 protein is expressed in the surface cells of the cecum of WT mice (Figure 1) in a similar location to DRA (Talbot & Lytle, 2010). Despite this, there is little evidence NHE3 is involved in basal oxalate transport. Compared to the WT, both the secretory and absorptive flux of oxalate was lower in the NHE3 KO cecum by ~25%, (albeit this was statistically significantly for former only) and there was no change in the net oxalate flux. These reductions maybe explained in part by the lower G_T_, which was also observed in the distal ileum and discussed above. That baseline oxalate transport rates were not disturbed in the NHE3 KO cecum might suggest that a partnering between NHE3 and DRA is not required for oxalate absorption via DRA.

A role for NHE3 in oxalate transport rates of FSK/IBMX stimulated ceca is more clear. In WT cecum, cAMP stimulated net oxalate secretion through a statistically significant drop in the oxalate absorptive flux and a steady increase in the oxalate secretory flux (Figure 3a), documenting for the first time that cAMP induces net oxalate secretion by the murine cecum. The statistically significant reduction in the absorptive flux might be due to the endocytosis of DRA from the apical membrane upon induction of increased intracellular cAMP levels (Musch et al., 2009). These effects of cAMP were greatly muted in the NHE3 KO cecum (Figure 3b) suggesting NHE3 plays an important role through its interactions with DRA. However, we note that successful cAMP ‐stimulated endocytosis of DRA has been observed in NHE3 KO jejuna (Musch et al., 2009). We cannot exclude the possibility that PAT1 may be involved in the cAMP ‐stimulated oxalate secretion since there is evidence PAT‐1 participates in basal sulfate secretion by the mouse cecum (Whittamore et al., 2013), and recent studies have shown FSK/IBMX can stimulate Cl^−^‐driven oxalate uptake in Caco‐2‐BBE cells through protein kinase A, a response that involves recruitment of PAT1 (slc26a6), and to a lesser degree DTDST (Diastrophic Dysplasia Sulfate Transporter, Slc26a2) (Arvans et al., 2020). DTDST is present in mouse large intestine (Park et al., 2014) but it is not known whether it is specifically expressed in the cecum. Additionally, cAMP has been shown to induce oxalate secretion in rabbit colon and ileum (Freel et al., 1998; Hatch, Freel, & Vaziri, 1993, 1994b) characterized as being mediated by an apical conductance (Freel et al., 1998) such as the cAMP‐activated Cl^−^ channel, CFTR. Expression studies in *Xenopus* oocytes have shown that human CFTR itself does not transport oxalate (Freel & Hatch, 2008; Knauf et al., 2017) but when co‐expressed with PAT1, either in the presence or absence of IBMX/FSK, results in increased (PAT1 mediated) oxalate efflux (Knauf et al., 2017). PAT1, CFTR, and DRA have all been shown to be linked with NHE3, with the connecting proteins seemingly important for regulation of ion transport. NHE3 has been shown to interact with CFTR in a reciprocal fashion (Bagorda et al., 2002; Favia et al., 2006; Mizumori et al., 2008) and both of these have been shown to be linked to PAT1 and DRA through PDZ domain containing proteins such as the various NHERF proteins (Lamprecht & Seidler, 2006). NHERF proteins are required for various aspects of salt and water transport across the intestine and the importance of each one varies segmentally (Ghishan & Kiela, 2012; Kato & Romero, 2011; Seidler et al., 2009). Further probing would be required to identify the specific contributions of each of these proteins to cAMP ‐induced regulation of oxalate secretion and absorption in the murine cecum, however, this study shows that this process is at least partially dependent on the presence on NHE3.

Sodium fluxes across the murine cecum have not previously been well studied. To our knowledge, sodium transport has only previously been directly measured in the ceca of C3H·heJBir mice, showing substantial net absorption of 20.9 ± 2.0 µeq/cm^2^·h (Homaidan et al., 1999). In contrast, we observed negligible net transport (1.01 ± 0.99 (11) µmol/cm^2^·h) under basal conditions (Table 3). This is may reflect a strain related difference. There was no statistically significant difference in net sodium transport in NHE3 KO ceca although unidirectional sodium fluxes were lower in the NHE3 KO cecum, which may be related to the reduced G_T_ (Table 3). However, in the WT cecum, FSK/IBMX led to a rapid decrease in the sodium absorptive flux (by 42%) promoting net secretion (Figure 3c). The absence of this reduction in the NHE3 KO indicates a role for NHE3 in the basal sodium absorptive flux despite the lack of net sodium absorption.

Overall in the cecum, it appears that NHE3 is involved in cAMP‐mediated stimulation of oxalate secretion and inhibition of sodium and oxalate absorption.

### 4.4 The distal colon

There was a little evidence that NHE3 plays a role in oxalate fluxes by the distal colon as there were no significant differences between WT and NHE3 KO (Table 4) and oxalate fluxes in the WT distal colon were not sensitive to FSK/IBMX application (Figure 4a). Furthermore, basal sodium flux rates were not different in the NHE3 KO compared to the WT (Table 4). However, NHE3 appeared to be important for cAMP inhibition of sodium absorption and the increase in electrogenic anion secretion (indicated by I_sc_), both of which were absent in the NHE3 KO distal colon (Figure 4e and e).

In the distal colon, a significant portion of oxalate absorption has been shown to occur through DRA under symmetrical, short‐circuit conditions in vitro (Freel et al., 2013), and similar to the cecum, there does not appear to be a role for PAT1 or any other DIDS‐sensitive anion transporter (Whittamore & Hatch, 2020). However, oxalate secretion in the murine distal colon is dependent on carbonic anhydrase (Whittamore et al., 2015). This implies that oxalate transport may be sensitive to changes in intracellular pH which can be caused by the loss of NHE3 (Praetorius et al., 2000; Walker et al., 2008). Furthermore, in human colon origin Caco‐2BBE cells DRA and NHE3 are thought to be functionally coupled with involvement of carbonic anhydrase (Musch et al., 2009). However, the current study demonstrated little evidence that NHE3 is required for continued transcellular oxalate absorption through DRA or is involved in carbonic anhydrase‐dependent secretion in murine distal colon (Table 4). Interestingly, both DRA and PAT1 mRNA have previously been observed to be upregulated in NHE3 KO colon (Laubitz et al., 2008), but this did not appear to exert any obvious influence on oxalate transport. This interpretation may be confounded by the secondary effects of renal salt wasting and fluid loss in the NHE3 KO, causing RAAS activation. Rats with chronic renal failure exhibit losartan sensitive increases in oxalate secretion across the distal colon (Hatch & Freel, 2003b; Hatch et al., 1999) suggesting that the RAAS may play a role in directly controlling oxalate transport in the distal colon. Whether RAAS activation induces oxalate secretion in the murine distal colon has not yet been investigated.

FSK/IBMX did not stimulate oxalate secretion in the WT distal colon (Figure 4a), unlike the cecum, and is the only intestinal segment to not respond in terms of oxalate fluxes. This is in contrast to previous work on the rabbit distal colon which has shown increased oxalate secretion in response to elevated intracellular cAMP (Hatch et al., 1994b). As such, it would appear the mechanisms and signaling pathways for cAMP‐mediated oxalate transport in the murine distal colon are distinctly different from rabbit colon and even between different intestinal sections within the same species.

The WT distal colon exhibited a small net absorption of sodium comparable to previous observations (Charney et al., 2004). Sodium transport rates were not statistically significantly different between WT and NHE3 KO distal colon under basal conditions (Table 4); however, the colon also expresses the epithelial sodium channel (ENaC) which may in part compensate for sodium absorption in the absence of NHE3. NHE3 KO mice are known to have high serum aldosterone (Schultheis, Clarke, Meneton, Miller, et al., 1998). Aldosterone targets the colon through mineralocorticoid receptor (MR) to regulate sodium absorption through NHE3 and ENaC (Nakamura et al., 2018). The NHE3 KO mouse has upregulated expression of the β and γ subunits of ENaC (Schultheis, Clarke, Meneton, Miller, et al., 1998) as do rats with chronic renal failure (Hatch & Freel, 2008). Further probing with amiloride to inhibit ENaC may be useful to determine the full extent to which either NHE3 or ENaC are contributing to sodium transport under basal Ussing chamber conditions, however this question was beyond the scope of this study with insufficient mice simultaneously available to investigate it. Despite remaining questions about the relative contributions of NHE3 and ENaC to basal sodium transport, this study still reveals that NHE3 is critical to regulation of sodium transport through cAMP in the murine distal colon. Upon addition of FSK/IBMX net sodium absorption was abolished through a reduction of the absorptive sodium flux in the WT (Figure 4c) with no corresponding reduction in the NHE3 KO distal colon (Figure 4d), consistent with cAMP inhibition of sodium absorption occurring predominantly through endocytosis of NHE3 (Chow et al., 1999). Furthermore, even if ENaC is providing some compensatory regulation in basal sodium transport, it does not appear to compensate for the loss of NHE3 in cAMP regulation of sodium transport.

Interestingly, NHE3 appeared necessary for cAMP induction of electrogenic anion secretion. FSK/IBMX increased I_sc_ in the WT distal colon but not the NHE3 KO (Figure 4e). As such, it would appear NHE3, or more likely its association to other electrogenic transporters such as CFTR has a key role in cAMP induced anion secretion. Although both CFTR and NHE3 are expressed in the distal colon, it is not clear how much their expression overlaps along the surface–crypt axis in the distal colon since CFTR is primarily located in the crypts (Jakab et al., 2011) whereas NHE3 expression was restricted to surface cells (Figure 1) consistent with previous reports (Talbot & Lytle, 2010). It is therefore not clear if CFTR and NHE3 are capable of physically interacting for the latter to influence the functioning of CFTR in response to cAMP in the native epithelium and thus requires further investigation.

## 5 CONCLUSIONS

The goal of this study was to assess whether NHE3 is involved in oxalate transport through its functional and regulatory interactions with the apical oxalate transporters PAT‐1 and DRA in the mouse intestine and how this might impact oxalate renal handling. Urinary oxalate excretion was approximately doubled in NHE3 KO mice, when expressed as a ratio of creatinine; however, this is similar to other models of impaired renal function which raises the issue that the kidneys may be able to adaptively maintain or increase urinary oxalate excretion and this likely does not occur through NHE3. In the intestine, NHE3 was not essential to sustain basal oxalate transport in any segment, although interactions between the RAAS may have impaired the ability to detect a difference in the distal colon.

A key finding was distinct segmental heterogeneity in terms of the response to cAMP. NHE3 appeared to play the biggest role in the cecum where cAMP ‐stimulated net oxalate secretion was considerably muted in the NHE3 KO. Additionally, NHE3 appeared to be critical for cAMP stimulation of electrogenic anion secretion by the distal colon, but not other intestinal sections examined. This study is also significant in demonstrating that cAMP can elicit oxalate secretion to varying degrees in the distal ileum and cecum, but not the distal colon.

## CONFLICTS OF INTEREST

The authors have no conflicts of interest, financial or otherwise to declare.

## AUTHOR CONTRIBUTIONS

C.E.S., J.M.W., and M.H. conceived and designed the research; C.E.S. performed thr experiments; C.E.S. and J.M.W. analyzed the data; C.E.S., J.M.W., and M.H. interpreted the results of experiments; C.E.S. prepared the figures; C.E.S. drafted the manuscript; C.E.S., J.M.W., and M.H. edited and revised the manuscript; C.E.S., J.M.W., and M.H. approved the final version of the manuscript.
